# Triggering receptor expressed on myeloid cells-1 (TREM-1): a new player in antiviral immunity?

**DOI:** 10.3389/fmicb.2014.00627

**Published:** 2014-11-26

**Authors:** Kelsey Roe, Sébastien Gibot, Saguna Verma

**Affiliations:** ^1^Department of Tropical Medicine, Medical Microbiology and Pharmacology, John A. Burns School of Medicine, University of Hawaii at ManoaHonolulu, HI, USA; ^2^Service de Réanimation Médicale, University Hospital of NancyNancy, France

**Keywords:** TREM-1, innate immune response, virus pathogenesis, inflammation, antiviral immunity

## Abstract

The triggering receptor expressed on myeloid cells (TREM) family of protein receptors is rapidly emerging as a critical regulator of a diverse array of cellular functions, including amplification of inflammation. Although the ligand(s) for TREM have not yet been fully identified, circumstantial evidence indicates that danger- and pathogen-associated molecular patterns (DAMPs and PAMPs) can induce cytokine production via TREM-1 activation. The discovery of novel functions of TREM, such as regulation of T-cell proliferation and activation of antigen-presenting cells, suggests a larger role of TREM proteins in modulation of host immune responses to microbial pathogens, such as bacteria and fungi. However, the significance of TREM signaling in innate immunity to virus infections and the underlying mechanisms remain largely unclear. The nature and intensity of innate immune responses, specifically production of type I interferon and inflammatory cytokines is a crucial event in dictating recovery vs. adverse outcomes from virus infections. In this review, we highlight the emerging roles of TREM-1, including synergy with classical pathogen recognition receptors. Based on the literature using viral PAMPs and other infectious disease models, we further discuss how TREM-1 may influence host-virus interactions and viral pathogenesis. A deeper conceptual understanding of the mechanisms associated with pathogenic and/or protective functions of TREM-1 in antiviral immunity is essential to develop novel therapeutic strategies for the control of virus infection by modulating innate immune signaling.

## Introduction

The robust induction of innate immunity is the first line of defense against virus infections and depends on the ability of the host immune cells to detect invading pathogens and alert adaptive immune cells. Viral pathogens and replicative intermediates present pathogen-associated molecular patterns (PAMPs), such as viral double-stranded (ds) RNA, uncapped single-stranded (ss) RNA and viral proteins, which are detected by pattern recognition receptors (PRRs) expressed on immune cells. Typically, binding of virus PAMPs to PRRs namely Toll-like receptors (TLRs), RIG-I-like receptors (RLRs) and NOD-like receptors (NLRs) cause activation of downstream signaling resulting in the production of antiviral type I interferon (IFN) and pro-inflammatory cytokines (Kumar et al., 2011). A timely production of IFN-α/β and pro-inflammatory cytokines is critical for the clearance of the virus during early phase of the infection and fine-tuning the innate-adaptive interface for long-lasting protection. The activation of these PRRs is very tightly regulated at multiple steps to prevent tissue damage due to uncontrolled production of cytokines. Thus, overall virus disease outcome depends on the magnitude and the nature of innate immune events triggered following virus entry. Understanding the host factors that participate in positive or negative regulation of innate immunity to virus infections is currently the focus of intense research to seek out cues for designing therapeutics to modulate inflammation at the molecular level. Several novel innate immune molecules, including members of triggering receptors expressed on myeloid cells (TREM) family of receptors, have been recently characterized to play an important role in modulating the intensity of innate immune responses.

Since the discovery of TREM in 2000, they have been described as critical immunomodulators in several inflammatory disorders of both infectious and non-infectious etiology (Ford and McVicar, 2009). TREM-1 is the most well-characterized member of the TREM family and plays an important role in the amplification of inflammation, crosstalk with other PRR pathways and activation of antigen-presenting cells (APC). A substantial amount of data supports the link between TREM-1 signaling and several diseases, such as polymicrobial septic shock and inflammatory bowel disease, and in animal models of pneumonia and asthma (Ford and McVicar, 2009; Klesney-Tait et al., 2012). Although the data on the functions of TREM-1 in virus infections is limited, *in vitro* studies using viral PAMPs suggest the ability of viruses to modulate TREM signaling. In this review, we first discuss the current understanding of the immunoregulatory functions of the TREM family, in particular TREM-1, and then highlight potential roles of TREM-1 in antiviral immunity.

## TREM family of receptors

Members of the TREM family (designated TREM-1 to TREM-4) belong to the immunoglobulin variable (IgV) domain receptor superfamily of proteins (Bouchon et al., 2000). They are cell surface activating receptors with a transmembrane region containing charged lysine residues and a short cytoplasmic tail lacking signaling motifs (Bouchon et al., 2000). Signaling through these receptors is facilitated by the adaptor protein DNAX-activating protein of 12 kDa (DAP12) (Bouchon et al., 2000). TREM-1 was designated as CD354 by the Ninth Workshop on Human Leukocyte Differentiation Antigens in 2011 (Matesanz-Isabel et al., 2011).

The human TREM family members, which share low sequence homology with each other, are located on chromosome 6p21.1 (Radaev et al., 2003; Kelker et al., 2004b) and cluster with the related NKp44 receptor (Allcock et al., 2003). Several related receptors, such as the TREM-like transcripts (TLT) 1-5 also map to this region of the human genome and share the V-domain of the Ig superfamily but unlike TREM, they also contain an immunoreceptor tyrosine inhibitory motif in their cytoplasmic tail (Allcock et al., 2003). In the laboratory mouse, the TREM genes map to chromosome 17C3 (Chung et al., 2002; Watarai et al., 2008; Paradowska-Gorycka and Jurkowska, 2013). TREM-3, which is not found in humans and shares 43% sequence similarity with TREM-1, is located directly next to TREM-1 in the mouse genome, and is predicted to be functionally similar to TREM-1 (Chung et al., 2002; Radaev et al., 2003). pDC-TREM (also called TREM-4 and not expressed in human cells) is present on mature mouse plasmacytoid dendritic cells (pDCs) and share ~20% amino acid homology with TREM-1 and TREM-2 (Watarai et al., 2008). Two independent groups have solved the structure of the extracellular IgV domain of TREM-1. Although, the results have been conflicting, both groups have validated that TREM-1 belongs to the Ig superfamily (Radaev et al., 2003; Kelker et al., 2004a,b).

## Cellular localization

TREM-1 and TREM-2 were first identified on lipopolysaccharide (LPS)-stimulated monocytes and neutrophils (Bouchon et al., 2000), following which the initial search for TREMs was mostly limited to immune cells. The types of human immune cells expressing high levels of TREM-1 includes monocytes, neutrophils, granulocytes, DCs and natural killer (NK) cells, and low level expression on T cells and all subsets of B cells (Allcock et al., 2003; Matesanz-Isabel et al., 2011; Rigo et al., 2011). However, more recent studies have characterized the expression of TREM family members in several human and mouse non-immune cells and tissues. Studies describing the presence of TREMs in different cell types and species are summarized in Table 1. The presence of TREM-1 is now reported in varying human and mouse non-immune cells such as epithelial cells and fibroblasts, and in tissues such as lymph nodes, spinal cord, lung, heart, and placenta (Gingras et al., 2002; Chen et al., 2008; Wu et al., 2012; Zangi et al., 2012). TREM-2 has the widest range of expression so far and includes myeloid cells, osteoclasts and microglia, and tissues such as kidney, liver, heart, brain, and lung (Schmid et al., 2002; Paloneva et al., 2003). As seen in Table 1, the literature on the expression patterns of other TREM and TLT family members is limited and mostly reports presence on mouse immune cells. However, this expanding list of cell types and species is likely to continue growing as further research on TREMs is undertaken.

**Table 1:** **Cellular expression profiles of the TREM family proteins**.

**Cell type/tissue**	**Species**	**References**
**TREM-1**		
Neutrophils, CD14^high^ Monocytes	Human	Bouchon et al., 2000
NK cell line, fibrosarcoma (HT1080)	Human	Allcock et al., 2003
Differentiated U937 cells	Human	Gingras et al., 2002
Lymph nodes, placenta, spinal cord, lung, spleen, and heart tissues	Human	Gingras et al., 2002
High expression: Monocytes, granulocytes, DCs, NK cell	Human	Matesanz-Isabel et al., 2011
Low expression: T cells and all subsets of B cells except plasma cells		
Normal bronchial epithelial cells	Human	Rigo et al., 2011
Myofibroblasts and primary hepatic stellate cells	Human	Liao et al., 2012
Gastric epithelial cell lines	Human	Schmaußer et al., 2008
Liver endothelial cells	Mouse	Chen et al., 2008
Kupfer cells and neutrophils	Mouse	Wu et al., 2012
Peritoneal macrophages	Mouse	Bouchon et al., 2001a
Immature dendritic cells	Mouse	Zangi et al., 2012
**TREM-2**		
Monocyte derived dendritic cells	Human	Bouchon et al., 2001a
NK cell line, fibrosarcoma (HT1080), U937, and THP-l	Human	Allcock et al., 2003
Activated macrophages, peritoneal macrophages, RAW264 cells	Mouse	Turnbull et al., 2006
Ostoclasts	Human	Paloneva et al., 2003
Microglia	Mouse	Schmid et al., 2002
High expression in CNS, heart and lungs as compared to lymph nodes, kidney, liver, and testes	Mouse	Schmid et al., 2002
**TREM-3**		
RAW264, MT2 macrophage cell lines, and T cell lines	Mouse	Chung et al., 2002
**pDC-TREM (TREM-4)**		
pDCs (CD11c^dull^ population)	Mouse	Watarai et al., 2008
**TLT-1**		
Platelets and megakaryocytes	Mouse	Washington et al., 2004
**TLT-2**		
B cells, neutrophils, and macrophages	Mouse	King et al., 2006

## Soluble TREM as marker of disease severity

Although TREM family members are typically membrane-bound receptors, multiple members are now reported to exist in a soluble form in the clinical samples of patients from several inflammatory conditions. So far, soluble forms of TREM-1, TREM-2, and TLT-1 have been identified (Klesney-Tait et al., 2006). However, it is possible that other members of the family might also be recognized in this form. The soluble form of TREM-1, a 27 kDa glycosylated peptide, is most likely produced by the cleavage of the extracellular ectodomain of the membrane-bound form by matrix metalloproteinases (Gómez-Piña et al., 2007). Using *in vitro* time-course analysis, Gómez-Piña and colleagues demonstrated that increased levels of sTREM-1 correlated with decreased cell surface TREM-1 expression after 6 h of LPS stimulation of CD14^+^ monocytes (Gómez-Piña et al., 2007). It remains probable, however, that sTREM-1 may be produced through alternate pathways, such as alternate spicing (Gingras et al., 2002).

In clinical conditions, sTREM was first identified in the plasma of patients with sepsis (Gibot et al., 2004c) and in bronchial lavage specimens of pneumonia patients (Gibot et al., 2004a). Elevated levels of sTREM-1 have now been found in multiple infectious and chronic inflammatory diseases such as pneumonia, pleural effusion, intra-abdominal infections, inflammatory bowel disorders, inflammatory rheumatoid disorders, and lung cancer as reviewed previously (Barraud and Gibot, 2011). In addition to the serum, two studies reported increased sTREM-1 levels in cerebral spinal fluid (CSF) of bacterial meningitis patients implying that it may be a marker of differentiating bacterial vs. non-bacterial meningitis (Determann et al., 2006; Bishara et al., 2007). Although the sample size of non-bacterial cases was very small as compared to bacterial meningitis cases, nonetheless, these studies emphasize that TREM-1 signaling may be modulated during CNS infections. Initial interpretations of these data led to the proposal of developing sTREM-1 as a biomarker for the diagnosis of acute inflammatory diseases, such as septic shock. However, this observation was not supported by subsequent studies and is now considered as a possible indicator of increased severity of the disease (Determann et al., 2006; Bucova et al., 2012). At present, the function of sTREM is unknown, but based on the data on other soluble forms of membrane receptors such as ICAM-1 and VCAM-1 (Page and Liles, 2013), it is possible that sTREM-1 and sTREM-2 may negatively regulate TREM receptor signaling via neutralization of the respective ligands.

## TREM ligands

Identification of the ligands for any receptor is a crucial step in establishing a link between a signaling receptor and disease pathogenesis. The search for TREM family ligands has been elusive, although multiple putative ligands have been proposed. Haselmayer and co-workers identified a membrane-bound ligand for TREM-1 on the surface of platelets, which upon interacting with TREM-1, amplified LPS-induced neutrophil activation (Haselmayer et al., 2007). Evidence for the presence of a soluble ligand for TREM-1 comes from a study showing that exposure of human monocytes to the serum of septic patients and LPS increased production of tumor necrosis factor-α (TNF-α), which was blocked by TREM-1/Fc fusion peptide (Wong-Baeza et al., 2006). Moreover, a few DAMPs and PAMP are described as possible TREM-1 ligands, indicating that TREM-1 could act as a PRR in a similar capacity as TLRs. Mohamadzadeh and colleagues used a viral replicon system to demonstrate that the surface glycoprotein, but not the nuclear protein, of Marburg virus was able to bind to a TREM-1/Fc fusion peptide (Mohamadzadeh et al., 2006). In the list of potential DAMPs, two independent studies have proposed high-mobility group box 1 protein (HMGB1) or heat shock protein 70 (HSP70) as possible ligands for TREM-1 (El Mezayen et al., 2007; Wu et al., 2012). HMGB1 and HSP70 present in the necrotic cell lysates of myeloid cells were responsible for significant induction of the proinflammatory cytokine expression, which was reduced by blocking TREM-1, thus confirming the role of TREM-1 in cytokine expression cascade via responding to these endogenous DAMPs. Collectively, these data suggest that TREM-1 may not have a single ligand, as most activating receptors do, but might recognize multiple epitopes and bind to a range of viral ligands.

## TREM-1 signaling and inflammation

Activation of TREM-1 signaling is initiated upon binding of the ligand to the receptor, which triggers the association and phosphorylation of immunoreceptor tyrosine-based activation motif of the adaptor protein DAP12. The signaling pathways triggered downstream to DAP12 phosphorylation are very specific to the TREM family member. *In vitro* studies in relevant cell types such as neutrophils and macrophages demonstrate that phosphorylation of DAP12 by Src family kinases results in the recruitment and activation of non-receptor tyrosine kinase Syk. The Syk, in turn, activates the downstream signaling molecules including PI3K, PLCγ, ERK1/2, and MAP kinases that regulate NF-κB activation and expression of inflammatory genes in a cell-specific manner (Bouchon et al., 2000; Tessarz and Cerwenka, 2008; Ford and McVicar, 2009). Although the exact pathway remains unclear, TREM-1 signaling via phosphorylation of Syk also regulates calcium influx, which further activates MAPK/ERK pathway (Arts et al., 2013). In neutrophils, TREM-1 also regulates neutrophil degranulation and production of reactive oxygen species in addition to cytokines and chemokines (Bouchon et al., 2001a; Radsak et al., 2004; Haselmayer et al., 2007) On the other hand, activation of DAP12 by TREM-2 is shown to promote anti-inflammatory response in a cell-specific manner (Paradowska-Gorycka and Jurkowska, 2013). In contrast to TREM-1, TREM-2 signaling does not involve NF-κB translocation and is shown to induce DAP12-dependent calcium influx followed by activation of ERK and PI3K (Bouchon et al., 2001b). Although the overlap and cross-talk between TREM-1 and TREM-2 signaling pathway is not yet clear, several studies demonstrating downregulation of cytokines such as TNF-α by TREM-2 (Takahashi et al., 2005) emphasize opposing physiological roles of these two receptors.

Amplification of inflammation is the best-characterized function attributed to TREM-1. Because of the absence of a well-characterized ligand, an agonist antibody to TREM-1 is routinely used to over activate TREM-1 signaling. Bouchon and colleagues first showed that the key functional outcome of the artificial over-activation of TREM-1 receptor in monocytes was the increased production of cytokines, such as TNF-α, monocyte chemoattractant protein 1 (MCP-1) and interleukin-1β (IL-1β) following LPS treatment (Bouchon et al., 2000). Following this observation, several studies demonstrated the ability of TREM-1 to amplify inflammation during septic shock. Pharmacological inhibition of TREM-1 by the use of synthetic peptides or fusion protein repeatedly prevented hyper-responsiveness and death during various experimental septic shock models: endotoxemia in mice or monkeys (Bouchon et al., 2001a; Derive et al., 2014), bacterial pneumonia in rats (Gibot et al., 2006), polymicrobial peritonitis in rodents and pigs (Gibot et al., 2004b; Derive et al., 2013). By contrast, genetic invalidation of TREM-1 leads to contrasting results: while some report a decreased bacterial clearance and survival during Pneumococcal pneumonia and Klebsiella pneumonia liver abscesses in mice (Hommes et al., 2014; Lin et al., 2014), the opposite has been described in the setting of Leishmania major infection (Weber et al., 2014). Further, using siRNA silencing of TREM-1 in the mouse, Gibot and colleagues demonstrated the importance of the balanced activation of TREM-1 signaling during sepsis. This study showed that partial silencing of TREM-1 was protective during peritonitis, while complete silencing was lethal to septic mice (Gibot et al., 2007). This effect of TREM-1-dependent enhancement of inflammatory response is also observed in non-infectious disease models including hemorrhagic shock and pancreatitis (acute inflammation) and chronic inflammatory bowel diseases and inflammatory arthritis (Barraud and Gibot, 2011). TREM-1-deficient mice displayed significantly attenuated disease that was associated with reduced inflammatory infiltrates and diminished expression of pro-inflammatory cytokines, thus representing an attractive target for treatment of chronic inflammatory disorders (Weber et al., 2014). Such data are significant in suggesting that TREM-1 is not simply an inflammatory amplifier, but also plays a regulatory role in influencing the disease outcome.

It is now established that for the culmination of balanced and effective innate immunity, it is crucial to have crosstalk between multiple innate immune signaling pathways. Many recent studies support a model of synergy between TREM-1 and other PRRs, although the precise mechanisms are yet unclear. Available data indicates that the synergy between TLRs and TREMs might be at two levels. First, is the ability of TLR ligands to increase the mRNA expression of TREM-1, and second, the amplification of TLR-induced inflammatory response by TREM-1. Exposure of immune cells to several PAMPs, such as LPS (a TLR-4 ligand) and lipoteichoic acid (a TLR-2 ligand), and microbial pathogens such as *Pseudomonas aeruginosa* have shown to increase TREM-1 mRNA (Bleharski et al., 2003; Zeng et al., 2007; Zheng et al., 2010). In a TLR-2 dependent manner, soluble fungal antigens were shown to up-regulate the expression of TREM-1 transcripts in macrophages (Buckland et al., 2011). Similarly, expression of TREM-1 mRNA following activation of macrophages by LPS was dependent on the TLR-4/NF-κ B pathway (Zeng et al., 2007). The reverse was not true, TREM-1 signaling had no effect on TLR-4 expression. The mechanisms by which TREM-1 amplifies TLR-initiated inflammation are still being investigated. However, available data suggest that the synergy is at the level of NF-κB and IRAK-1 and appears to be cell-specific (Arts et al., 2013). Activation of TREM-1 using agonistic monoclonal antibody in combination with the ligands for TLR-4 was shown to synergistically amplify the production of proinflammatory cytokines in monocytes (Bouchon et al., 2000). More recent studies by Ornatowska and colleagues used pathway-specific microarray analysis to show that TREM-1 silencing did not alter expression of TLR-4, but reduced the expression of adaptor protein Myd88 and cytokines such as IL-1b and IL-10, thus emphasizing that regulating expression of downstream signaling molecules may be one of the mechanisms of TREM-1/TLR cross talk (Ornatowska et al., 2007). Likewise, Hu and group further support the role of MyD88 protein as the point of cross talk between TREM-1 and TLR-4 signaling in the infection of corneal epithelial cells with fungi *Aspergillus fumigatus* (Hu et al., 2014). Additionally, artificial activation of TREM-1 is also shown to down-regulate expression of Tollip and ST2, negative regulators of TLR-2 and TLR-4 pathways (Lagler et al., 2009; Wu et al., 2011) These studies collectively emphasize the fact that cross talk between TLRs and TREM-1 is at multiple levels.

Most of the TREM research so far has focused on non-viral infections and autoimmune diseases. However, characterization of several additional roles of TREM-1 such as modulation of T-cell proliferation and APC activation clearly argues for its crucial immunomodulatory role in virus infections. Below we present essential elements of antiviral immune responses and then discuss how TREM-1 signaling fits into these immune events based on the current literature on TREMs in innate-adaptive interface.

## Antiviral immunity

Immune responses to virus infections are as diverse and complex as the viruses that induce them. However, there are specific events shared by many viruses, which are important determinants of virus clearance vs. immunopathology. An important feature of an efficient innate response to the entry of both DNA viruses, such as herpes simplex virus and cytomegalovirus, and RNA viruses, including Influenza A virus, West Nile virus (WNV) and chikungunya virus (CHIKV), is the rapid detection by PRRs, notably endosomal TLRs (TLR-3, TLR-7/8 and TLR-9), RLRs (RIG-I and MDA5) and the NLRs (NLRP3 and NOD2) (Lund et al., 2004; Wang et al., 2004; Varani et al., 2007; Zhang et al., 2007; Daffis et al., 2008; Kumar et al., 2013). The production of inflammatory cytokines is one of the hallmarks of PRR activation in innate immune cells including dendritic cells (DCs), and is required for the recruitment and activation of inflammatory cells such as macrophages, NK cells and neutrophils to the site of infection (Saitoh et al., 2012; Jenne et al., 2013). The profile of cytokines produced by innate immune cells dictates the adaptive immune response and the virus disease outcome. Unlike bacterial infections, another major innate immune response to virus infection is the production of type I IFN. The paracrine and autocrine secretion of IFN renders cells “antiviral” by inducing several interferon-stimulated genes (ISGs). These ISGs confer an antiviral state by blocking virus replication at different levels such as early-stage virus infection, inhibition of post-transcriptional modification and virus maturation, activation of macrophages and DC and stimulation of NK cells to kill virus-infected cells.

As an immediate effect of innate immune activation, effector cells such as NK cells and CD8^+^ T cells are recruited at the site of infection. These cells act to kill virus-infected cells and macrophages clear the resulting debris. Further, depending on the cytokines induced by the APCs, different types of T helper cell responses are induced. Recruited CD4^+^ T cells progress toward a T_H_1 phenotype in most viral infections, eventually leading to the induction of several components of adaptive immunity. However, viruses such as human immunodeficiency virus type 1 (HIV-1), HSV and hepatitis C virus (HCV) also drive T_H_17 and T regulatory cells (T_REG_) cell expansion (Rouse and Sehrawat, 2010). Influenza virus is another example where T_H_17 cell responses mediate recruitment of neutrophils, which then become responsible for the associated lung pathology (Bermejo-Martin et al., 2009). Humoral immunity provided by specific neutralizing antibodies is also an essential component of the adaptive immune response to virus infection that inhibits virus attachment, internalization and protection against subsequent infection. At the later stages of infection, resolution of inflammation and the return to homeostasis is mediated by anti-inflammatory components, classically T_REG_ and the cytokines IL-10 and TGF-β, to prevent tissue damage after virus clearance. Viruses, such as HSV, HCV, and HIV use the strategy of increased T_REG_ functions to facilitate persistent infection (Veiga-Parga et al., 2013). Thus, induction of an effective and balanced innate immune response is an important determinant of virus disease outcome and is fine-tuned by multiple immune components. Understanding the specific mechanisms associated with the fine control of innate immune signaling pathways has been greatly enhanced because of the identification of several novel host molecules involved with either blocking or facilitating the synergy between important immune signaling pathways. TREMs family of proteins represents this class of novel innate immune molecules, which can influence the innate immune responses to viruses. The potential roles of TREM-1 in the different arms of viral immunity are discussed in the sections below.

## Activation of TREM-1 signaling by viruses

In 2006, Mohamadzadeh and colleagues first reported activation of TREM-1 signaling in filovirus-infected neutrophils. Although Marburg and Ebola viruses do not replicate in primary human neutrophils, they increased TREM-1 expression following internalization, which correlated with phosphorylation of DAP12 and ERK1/2. Additionally, this study also suggested that the surface glycoprotein (GP) of filoviruses may act as a ligand for TREM-1 (Mohamadzadeh et al., 2006). In the clinical scenario, increase in the neutrophils has been reported during the human Ebola disease (Martini, 1973; Fisher-Hoch et al., 1985). Further, *in vitro* studies have demonstrated that the soluble variant of Ebola GP can interact with neutrophils (Yang et al., 1998). Therefore, it is possible that the interaction between TREM-1 on neutrophils with the GP protein during infection contributes to the “cytokine storm” associated with lethal filovirus disease (Wauquier et al., 2010). Similarly, exposure of PBMC to the gp41 protein of HIV-1 has been shown to up-regulate the mRNA expression of TREM-1 (Denner et al., 2013). Another recent study by Suthar and co-workers used transcriptional profiling and pathway modeling to show that TREM-1 signaling was enriched in the liver following infection with WNV in mice (Suthar et al., 2013). Although this study implies that TREM-1 signaling might be one of the pathways responsible for restricting tissue tropism, the precise role of TREM-1 in WNV pathogenesis has not been explored.

However, indirect evidence supports the ability of viruses to induce TREMs. Bleharski and colleagues first showed that poly (I:C), a ligand for TLR-3, can induce the transcription of TREM-1 in primary monocytes (Bleharski et al., 2003). Later, studies by Begum and co-workers could not validate the increase of TREM-1 mRNA following stimulation of monocytes with poly (I:C), however the reason for this discrepancy might be the different time points of analysis, 6 h after stimulation, as compared to 24 h time point used in the previous study (Begum et al., 2004). Watarai and colleagues demonstrated that TREM-4 mRNA expression increased in mouse pDCs following TLR-7 or -9 activation and led to DAP12-phosphorylation, activation of ERK1/2 signaling and ultimately IFN-α secretion (Watarai et al., 2008). Although such studies are limited, they strongly suggest that diverse RNA and DNA viruses that produce poly (I:C) and CpG DNA may be capable of inducing TREMs.

Similarly, our understanding of whether viruses can induce production of sTREM-1 is unclear and so far comes from only one clinical study. Ruiz in-Pacheco and colleagues recently demonstrated increased levels of sTREM-1 in the serum of dengue virus (DENV)-infected patients during the early stages of infection (first 5 days) as compared to healthy individuals (Ruiz-Pacheco et al., 2014). At this point, one can only speculate the role of sTREM in pathogenesis of DENV, an important global human pathogen, however, this is an important finding and provides direct evidence of modulation of TREM-1 in response to virus infection. Elevated levels of sTREMs could signify either a virus-induced compensatory mechanism to counteract inflammatory process, or a host-induced mechanism to control tissue damage by attenuating downstream inflammatory signals. Therefore, such clinical studies will be highly relevant to identify the potential of sTREM-1 as a marker of disease severity in acute virus infections as with influenza virus and CHIKV.

## Impact of TREM-1 on virus-associated inflammation

**Figure 1:**
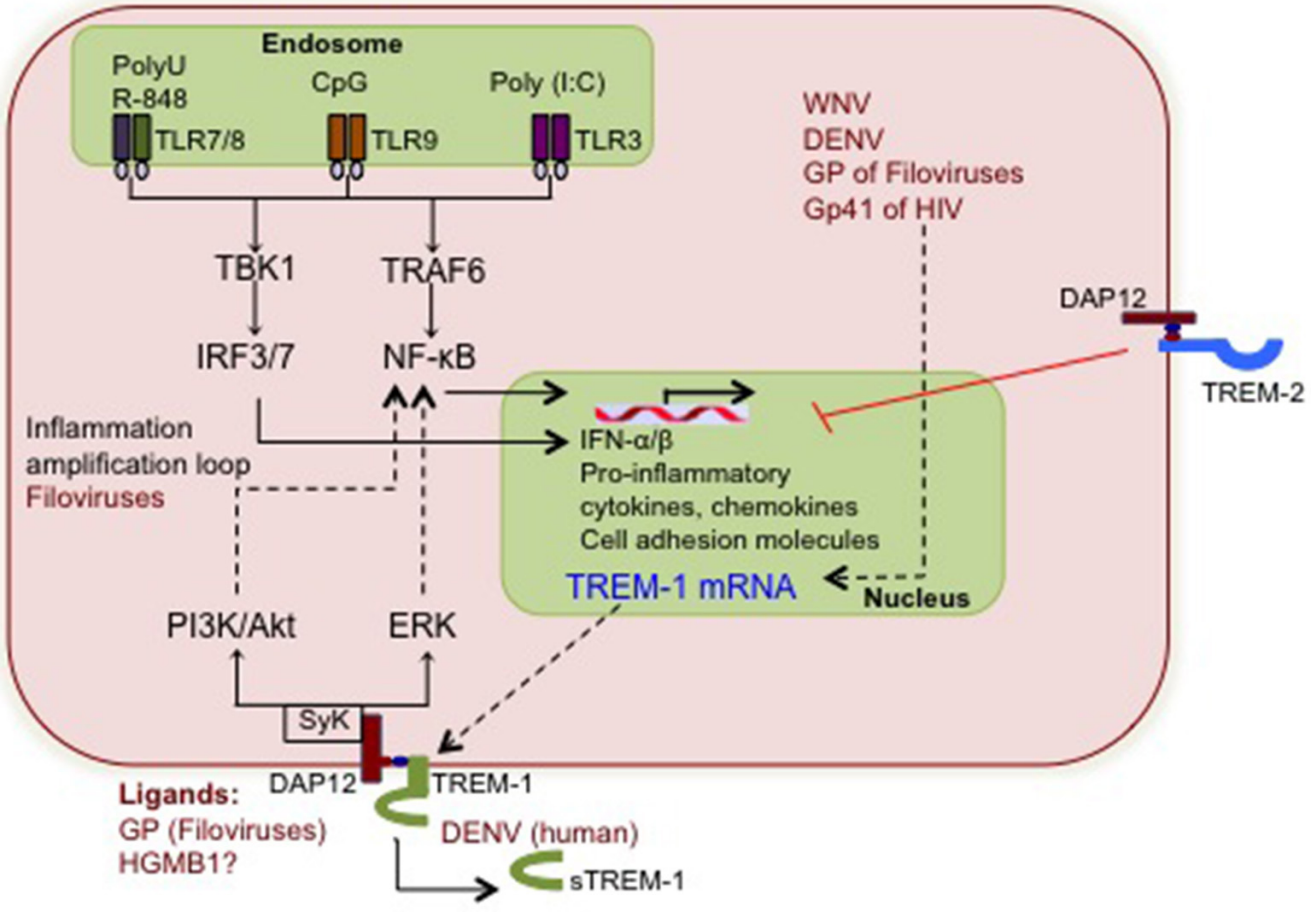
**The putative interactions between viruses and TREM-1 signaling**. During early stages of infection, viral nucleic acids and some proteins are detected by TLR-3, TLR-7/8 and TLR-9, which induce the mRNA production of pro-inflammatory cytokines, chemokines, and cell surface receptors including TREM-1. Viruses shown to increase TREM-1 mRNA and/or soluble TREM-1 levels are depicted in red, although the associated pathways are not clear. Increased TREM-1 receptor responds to yet uncharacterized viral or putative host ligands and activates signaling via DAP12 and Syk tyrosine kinase. Downstream PI3K and ERK signaling further activate NF-κ B and synergizes with the TLR cascade to amplify inflammation. The potential interactions between virus-induced TREM-1 and other cellular pathways such as type I IFN and apoptosis are not yet defined.

The function of TREM-1 in modulating virus-associated inflammation appears to be supported more by *in vitro* studies using viral PAMPs, than actual virus infections (Figure 1). Activation of TREM-1 by TLR-9 ligand CpG DNA enhanced TNF-α production in mouse bone marrow-derived dendritic cells (BMDCs) (Hara et al., 2007). Similarly, Netea and colleagues noted increased production of TNF-α in TREM-1-activated human PBMC following stimulation with poly (I:C) and CpG (Netea et al., 2006). This was also the first study to indicate that TREM-1 synergizes with NLR pathways. NLR ligands amplified the production of TNF-α, IL-1β, and IL-6 when TREM-1 signaling was activated (Netea et al., 2006). Similarly, TLR-TREM synergistic activation of neutrophils is not restricted to TLR-2 and TLR-4 but also occurs with TLR-7 and TLR-8, which are common TLRs responding to virus PAMPs (Radsak et al., 2004). Filoviruses are the only viruses, where the function of TREM-1 in regulating production of pro-inflammatory cytokines such as TNF-α and IL-1β is documented (Mohamadzadeh et al., 2006). The impact of TREM-1 in regulating inflammation in other virus diseases awaits discovery. Comprehensive analysis of virus infections *in vitro* as well as in TREM-1 deficient mice will be required before we fully understand the cooperation between TREM-1 and other viral PRRs in the context of the virus-associated inflammation.

## Modulation of type I IFN by TREM-1

The ability of the innate immune cells to rapidly produce type I IFN is one of the major determinants of the virus disease outcome. However, its role in other non-viral disease models, including bacterial infections, shock, and autoimmunity is less well-defined and complicated. With the emphasis of TREM-1 studies mainly in bacterial infection models, the role of TREM-1 in the regulation of type I IFNs has not been investigated so far. However, other TREM members have been shown to positively or negatively regulate type I IFN responses. In plasmacytoid DCs, stimulation of TREM-4 resulted in increased IFN-α secretion, and was dependent on the phosphorylation of DAP12 and PI3K and ERK1/2 pathways (Watarai et al., 2008). Conversely, mouse BMDCs deficient in TREM-2 had increased transcriptional levels of type I IFN following TLR-9 activation (Ito and Hamerman, 2012). Therefore, the fact that TREM-1 can synergize with TLR-3 and TLR-7 and signal through the ERK1/2 pathway leads us to speculate that TREM-1 might play a role in positively regulating type I IFN levels.

## Role of TREM-1 in APC activation, migration, and T-cell priming

The availability of recently developed TREM-1-deficient mice have led to studies describing several novel functions of TREM family members in addition to amplifying inflammation. Wu and colleagues developed a TREM-1 knockout (KO) mouse and demonstrated that TREM-1 was essential for the activation of Kupffer cells in the diethylnitrosamine model of hepatocellular carcinoma and contributed to chronic liver damage. TREM-1 KO macrophages were not as responsive as WT to signals from necrotic hepatocytes and exhibited attenuated APC responses including reduced production of IL-1β, IL-6, TNF-α, and CCL2 (Wu et al., 2012). In line with this, the role of TREM-1 in leukocyte recruitment is clearly demonstrated by Klesney-Tait and co-workers who used a double knockout TREM-1/3 mice for *Pseudomons aeruginosa* infection (Klesney-Tait et al., 2012). TREM-1/3 deficiency increased mortality of infected mice, which correlated with higher local and systemic cytokine production. However, although TREM-1/3-deficient neutrophils had intact bacterial killing and chemotaxis properties, histologic examination of TREM-1/3-deficient lungs revealed decreased neutrophil infiltration of the airways (Klesney-Tait et al., 2012).

Another novel function attributed to TREM-1 recently is its ability to influence the differentiation of primary monocytes into immature DCs. TREM-1 governs the upregulation of the surface expression of CD86 and MHC class II, rendering them more efficient at eliciting T-cell proliferative activity (Bleharski et al., 2003). Likewise, it has been demonstrated that signaling through TREM-1 on hypoxic iDCs up-regulates T cell co-stimulatory molecules, including CD83, CD86, and HLA-DR. Co-culture of these hypoxic iDCs with T-cells resulted in increased the cell proliferation and IFN-γ and IL-17 production (Pierobon et al., 2013). However, alternatively, Ito and Hamerman used TREM-2 deficient BMDCs to demonstrate that TREM-2 inhibited TLR-induced DC maturation and antigen presentation to T cells (Ito and Hamerman, 2012) thus supporting the dynamic opposite roles of TREM-1 and TREM-2 at the level of antigen presentation as well.

APC activation is essential during virus infection for the stimulation of T_H_1 responses and subsequent development of neutralizing antibodies vital for viral clearance. In addition to APC activation, TREM signaling also can skew T-cell responses in a T_H_1 or T_H_17 direction. Pierbon and colleagues, for instance showed that TREM-1 signaling in hypoxic iDCs was responsible for T_H_1/T_H_17 priming (Pierobon et al., 2013), while two independent studies showed a reduction in systemic T_H_1 responses following inhibition of TREM-1. In a model of *Pseudomonas aeruginosa-*induced keratitis, inhibition of TREM-1 reduced IFN-γ responses (T_H_1 phenotype), while increasing T_H_2 cytokines including IL-4 and -5 (Wu et al., 2011). Similar results were obtained in a model of cardiac allografts, in which TREM-1 inhibition led to increased allograft survival by dampening the differentiation and proliferation of IFN-γ secreting CD4+ T cells (Schiechl et al., 2013). In addition, TREM-1 signaling can be influenced by a T_H_1 environment; treatment of primary human NK cells with the classic T_H_1 cytokines IL-12 and -18 led to the activation of TREM-1 signaling events, as measured by microarray analysis (Grangeiro de Carvalho et al., 2011). Currently, there is no evidence supporting the hypothesis that TREM-1 may modulate innate-adaptive immune interface in virus infections. However, because viral ligands can activate TREM-1 signaling and cytokines production, there is high likely hood that cytokines governed by TREM-1 may influence activation of APCs, which in turn may impact protective T-cell functions and viral clearance.

## Return to homeostasis

Anti-inflammatory reactions are important in any immune reaction to return the host to homeostasis. In virus infections, one of the factors that determine the balance between virus clearance and tissue damage is the timely induction of anti-inflammatory molecules. Available data indicate that TGF-β and IL-10 treatment of primary monocytes synergistically down-regulates cell surface expression of TREM-1 (Schenk et al., 2005), but whether TREM-1 signaling can influence production of TGF-β and IL-10 has not been clearly defined. Nonetheless, this study strongly supports the notion that TREM-1 is the target of anti-inflammatory cytokines and that attenuating TREM-1- associated information might be one of the events in the immune homeostasis process. In microglia, TREM-2 participates in the process of tissue debris clearance and resolution of latent inflammatory reactions in Nasu-Hakola disease, a recessively inherited chronic neurodegenerative disease (Colonna, 2003). Similar anti-inflammatory and protective functions of TREM-2 have been proposed for other acute neuroinflammatory diseases, such as multiple sclerosis (Piccio et al., 2007) and Alzheimer's disease (Guerreiro et al., 2013; Jonsson et al., 2013), implying that alterations in the immune homeostasis may be mediated by TREMs in infections with neurotropic viruses, such as HIV, WNV or tick-borne encephalitis virus (TBEV).

## Conclusions and future perspectives

The immune functions of the TREM family are broad and diverse (Table 2) and may contribute to antiviral immunity. One of the pertinent questions is, can TREM-1 signaling respond to infection with globally important human viruses? Of particular interest would be the viruses associated with acute inflammation as the major cause of pathology such as orthomyxoviruses (influenza virus), filoviruses (Ebola and Marburg viruses), flaviviruses (DENV, WNV, and TBEV) and alphaviruses (CHIKV). More importantly, it is essential to characterize the role of TREM-1-dependent responses in disease outcome, i.e., protective vs. pathogenic. For example, it is likely that TREM-1 signaling may promote inflammation and cause substantial tissue damage thereby contributing to disease pathogenesis of acute infections as with influenza virus or CHIKV. In this regard, Weber and colleagues recently demonstrated that TREM-1 deficient mice were protected from severe influenza disease without affecting virus clearance, although this study did not look into the innate immune markers governed by TREM-1 (Weber et al., 2014). On the other hand, in virus infections with WNV, TBEV or Japanese encephalitis virus, activation of TREM-1 might be protective and enhance the robustness of innate immune responses in the periphery thereby facilitating efficient virus clearance and reduced neuroinvasion. This notion is supported by our unpublished data demonstrating increased mortality and morbidity in WNV-infected TREM-1/3-deficient mice. Our unpublished data further demonstrates that WNV can induce TREM-1 mRNA in a cell-type specific manner.

**Table 2:** **The role of the TREM family receptors in immune responses relevant to antiviral immunity**.

**Immune response**	**Function of TREMs**	**Model**	**References**
Inflammation/PRR signaling	Exposure to Poly(I:C) up-regulates trascription of TREM-1	Human monocytes	Bleharski et al., 2003; Netea et al., 2006
	TREM-1 synergizes with TLR-7/8	Neutrophils	Radsak et al., 2004
	TREM-1-TLR-9 (CpG) synergy amplifies TNF-α production	Mouse BMDC and human PBMC	Netea et al., 2006; Hara et al., 2007
	Crosstalk between NLR and TREM-1 signaling increases TNF-α, IL-Iβ and IL-6 production	Human PBMC	Netea et al., 2006
Type I IFN response	pDC-TREM (TREM-4) contributes to IFN-α production	Mouse splenocytes	Watarai et al., 2008
	TREM-2 has inhibitory action on type I IFN mRNA expression	Mouse BMDC	Ito and Hamerman, 2012
APC activation	TREM-1 modulates iDC differentiation and induce expression of CD1a, CD86, and HLA-DR	Human monocytes	Bleharski et al., 2003
	Deficiency of TREM-1 attenuates activation of Kupfer cells	*In vivo* mouse model	Wu et al., 2012
	TREM-1 promotes up-regulation of T-cell co-stimulatory molecules and production of pro-inflammatory mediators	Hypoxic iDC	Pierobon et al., 2013
	TREM-2 inhibits the induction of antigen-specific T-cell migration	Mouse BMDC	Ito and Hamerman, 2012
T_H_1 Response	IL-12 and -I8 treatment can induce TRE M-1 signaling	Human NK cells	Grangeiro de Carvalho et al., 2011
	TREM-1 promotes a T_H_1/T_H_17 response during hypoxic conditions	Hypoxic iDC	Pierobon et al., 2013
	Inhibition ofTREM-1 decreases the differentiation and proliferation of IFNγ producing CD4+ T cells	*In vivo* mouse model	Schiechl et al., 2013
	Inhibition of TREM-1 decreases T_H_1 responses and TLR signaling and increases T_H_ 2 cytokine production in infection	*In vivo* mouse model of *P. Aeruginosa*	Wu et al., 2011
Other elements of viral inflammution	TREM-1 mediates LPS-induced NOS-2 expression	Mouse macrophages and endothelial cells	Chen et al., 2008
	Exposure to Filoviruses led to TREM-1-dependent production of cytokines	Human neutrophils	Mohamadzadeh et al., 2006
	LPS-induced production of CCL2 is dependent on TREM-1	Human monocytes	Bouchon et al., 2000
Return to homeostasis	TGF-β and IL-10 treatment down-regulates TREM-1 expression	Human monocytes	Schenk et al., 2005

The question of how TREM-1 might influence disease outcome differently in acute vs. chronic infections with HIV or HCV also remains to be explained. Identification of inflammatory cytokines and chemokines regulated by TREM-1 will provide valuable insights into the role of TREM-1 in APC activation, T-cell responses and anti-viral immunity. Further, given that the type I IFN system is a powerful line of defense, characterization of the function of TREM-1 in modulating IFN levels and development of effective neutralizing antibody responses will further enhance our understanding of the complex network of antiviral immunity and fine-tuning of adaptive immunity.

In conclusion, this review describes the possible roles TREM-1 might play during virus infections. Future research using newly developed TREM-1 knockout mice and clinical samples from infected patients should focus on assigning protective or pathogenic functions for different viruses, examining the underlying cell- and tissue-type specific mechanisms of action, and identification of potential ligands responsible for TREM-1 receptor activation. There is such a large impact of the innate immune responses on the outcome of various viral diseases, therefore knowledge of the interactions between TREM-1 and viral PRRs (TLRs, NLRs, and RLRs) may lead to a better understanding of the pathophysiology of viral diseases. Further advancement in this field is crucial before TREM-1 can be proposed as an immunotherapeutic target to ultimately promote virus clearance with minimum tissue damage.

### Conflict of interest statement

The authors declare that the research was conducted in the absence of any commercial or financial relationships that could be construed as a potential conflict of interest.
